# Supplementary material for: Early Postnatal Outcome and Care after in Utero Exposure to Lithium: A Single Center Analysis of a Belgian Tertiary University Hospital
Source: Int J Environ Res Public Health. 2022 Aug 16;19(16):10111. doi: 10.3390/ijerph191610111 (PMC9407712; doi:10.3390/ijerph191610111)
Supplement: Supplementary file 1 [file ijerph-19-10111-s001.zip › ijerph-1822519-supplementary.pdf]

Supplementary Figures

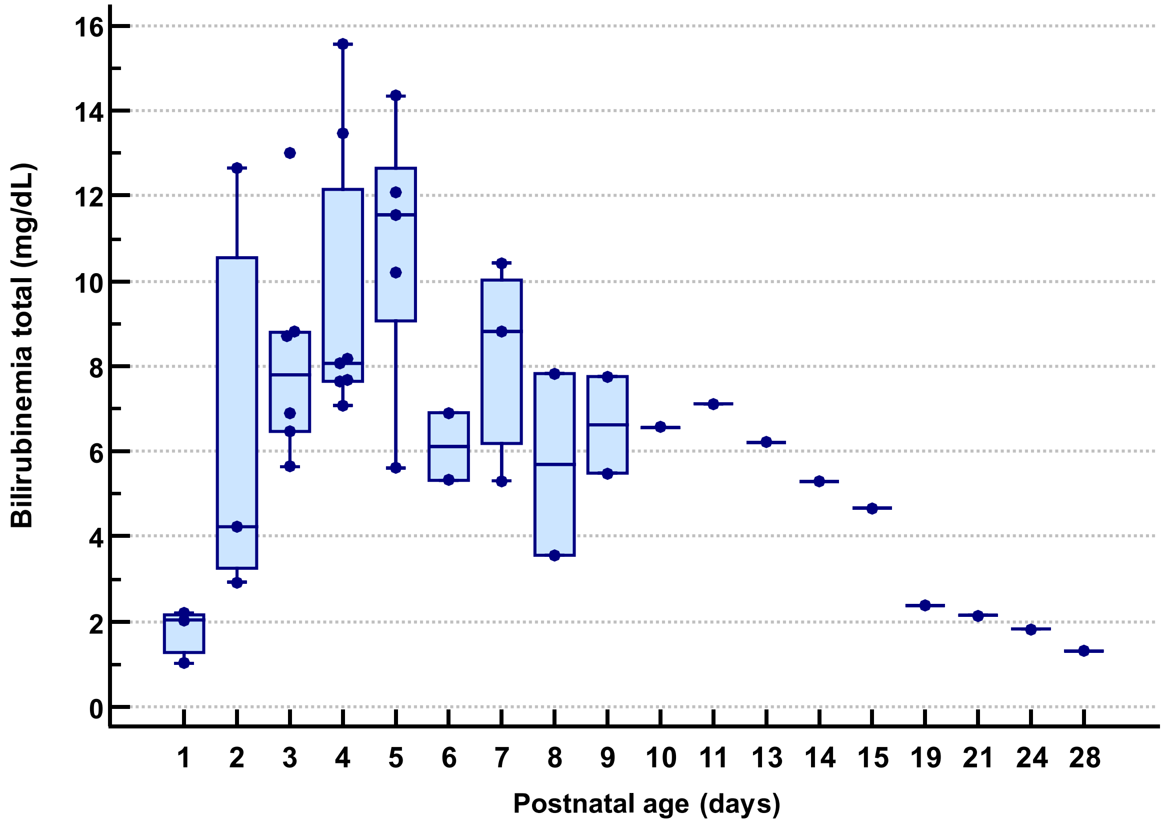

Figure S1: Available total bilirubinemia (mg/dL) data of the study population during the neonatal period (postnatal age day 1-28) are presented as boxplots. The dots represent all individual measurements. The x-axis indicates each postnatal day on which total bilirubinemia observations are available. From day 10 to 28, all data originate from 1 patient.

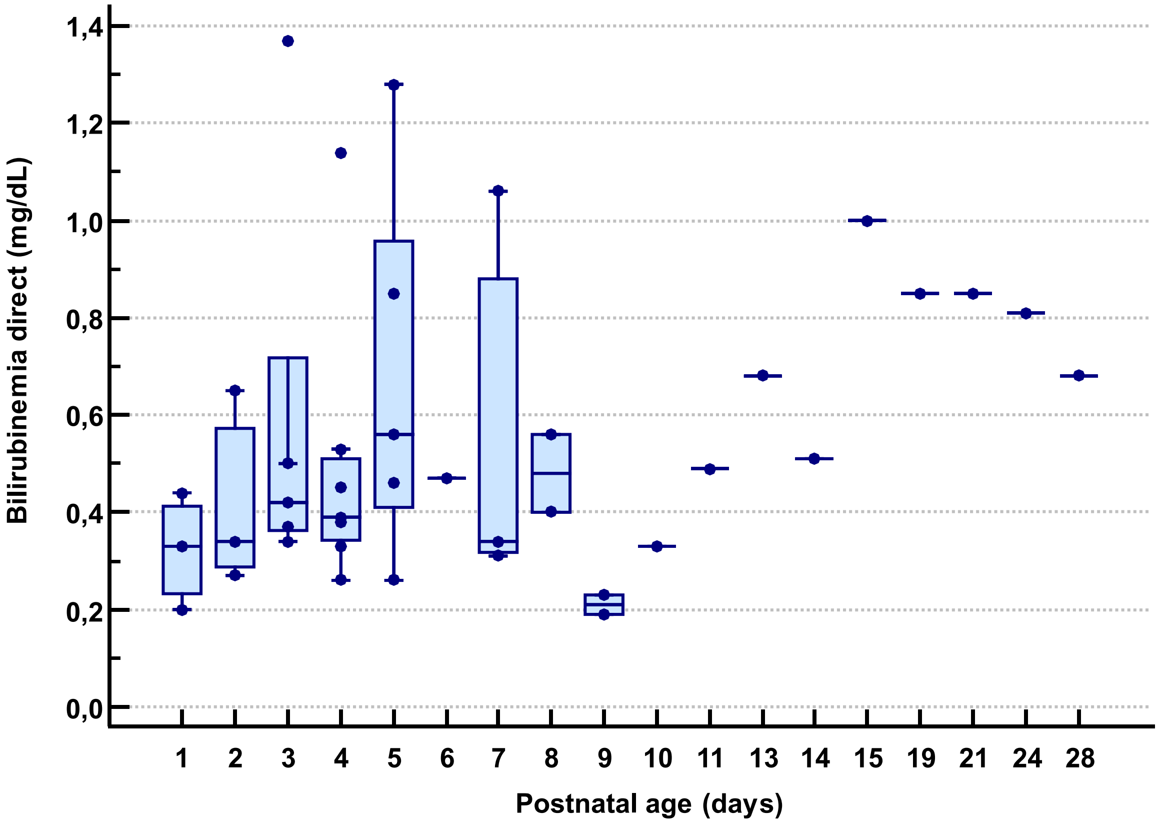

Figure S2: Available direct bilirubinemia (mg/dL) data of the study population during the neonatal period (postnatal age day 1-28) are presented as boxplots. The dots represent all individual measurements. The x-axis indicates each postnatal day on which direct bilirubinemia observations are available. From day 10 to 28, all data originate from 1 patient.

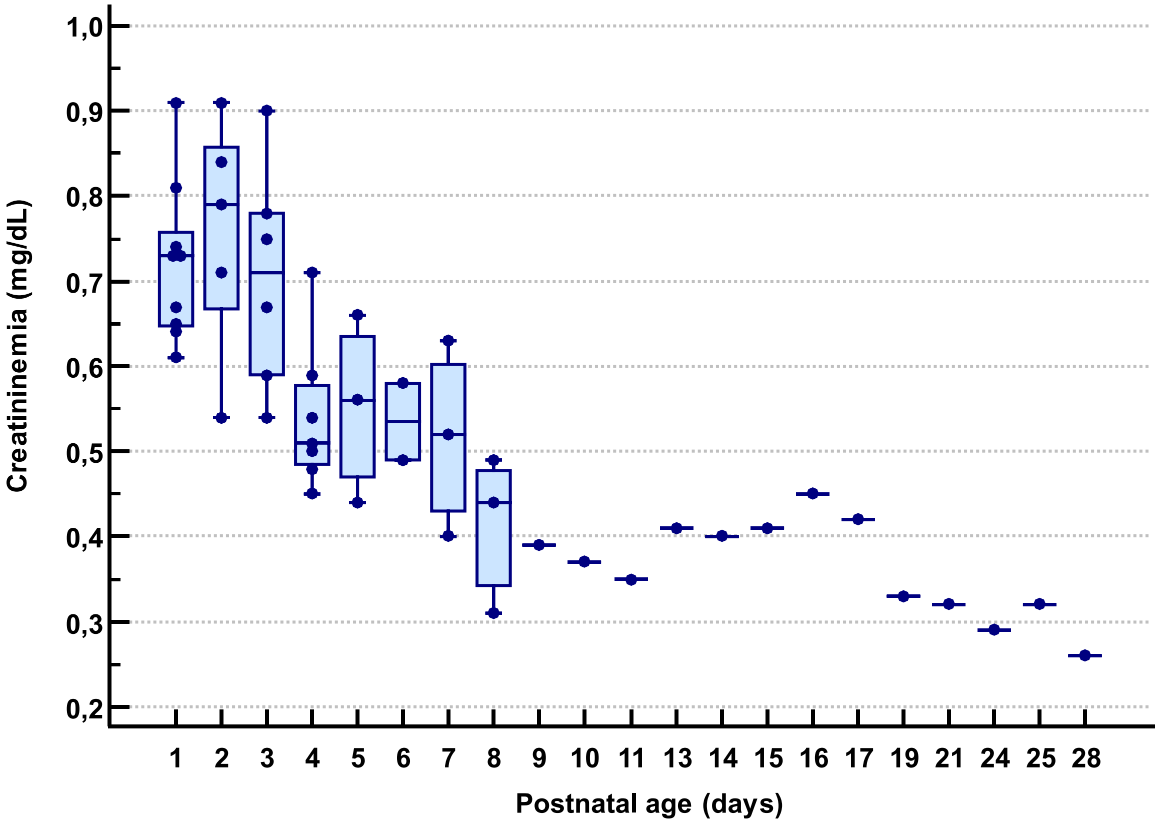

Figure S3: Available creatininemia (mg/dL) data of the study population during the neonatal period (postnatal age day 1-28) are presented as boxplots. The dots represent all individual measurements. The x-axis indicates each postnatal day on which creatininemia observations are available. From day 9 to 28, all data originate from 1 patient.

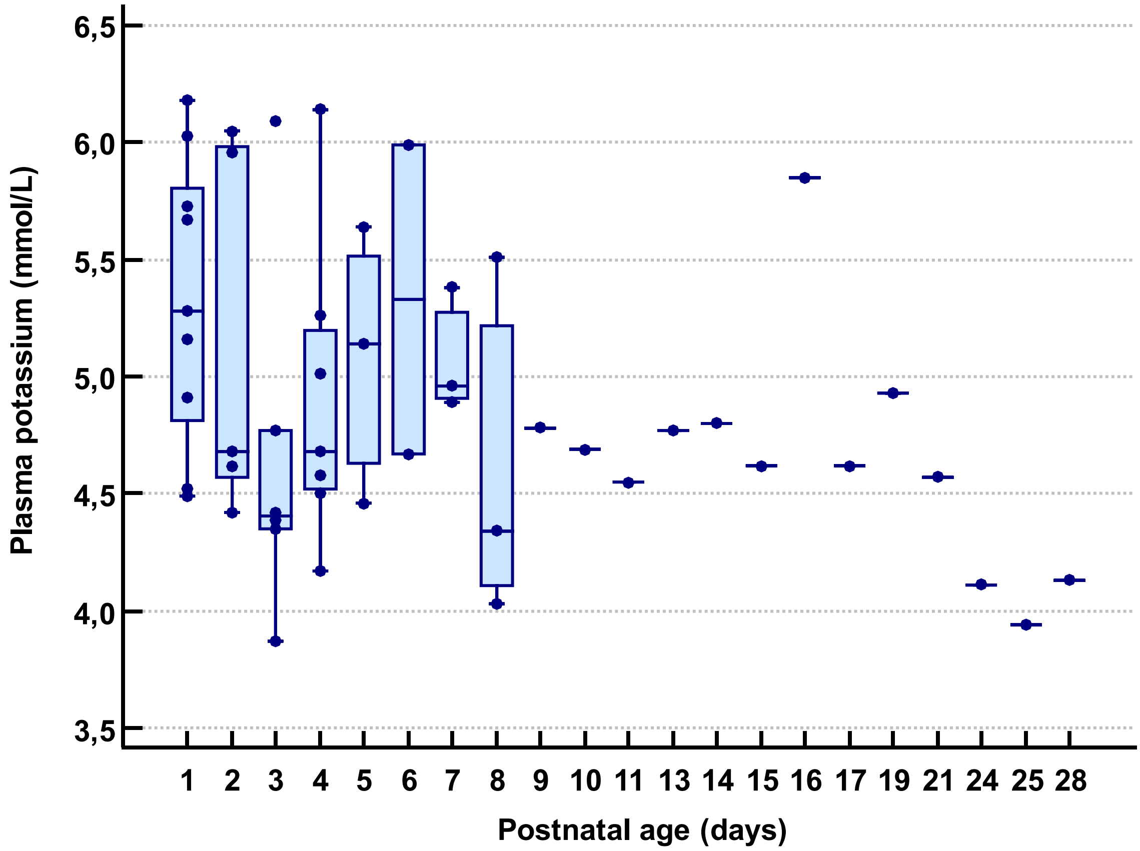

Figure S4: Available plasma potassium data of the study population during the neonatal period (postnatal age day 1-28) are presented as boxplots. The dots represent all individual measurements. The x-axis indicates each postnatal day on which potassium observations are available. From day 9 to 28, all data originate from 1 patient.

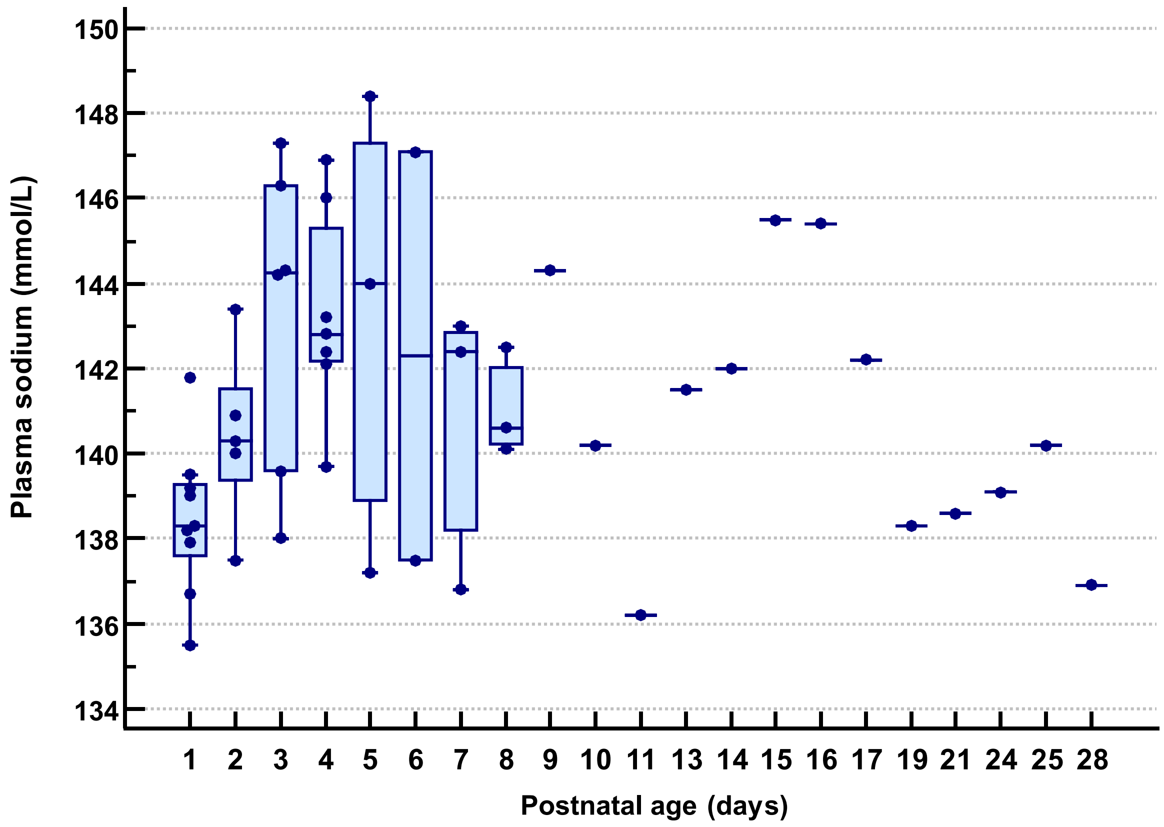

Figure S5: Available plasma sodium data of the study population during the neonatal period (postnatal age day 1-28) are presented as boxplots. The dots represent all individual measurements. The x-axis indicates each postnatal day on which sodium observations are available. From day 9 to 28, all data originate from 1 patient.

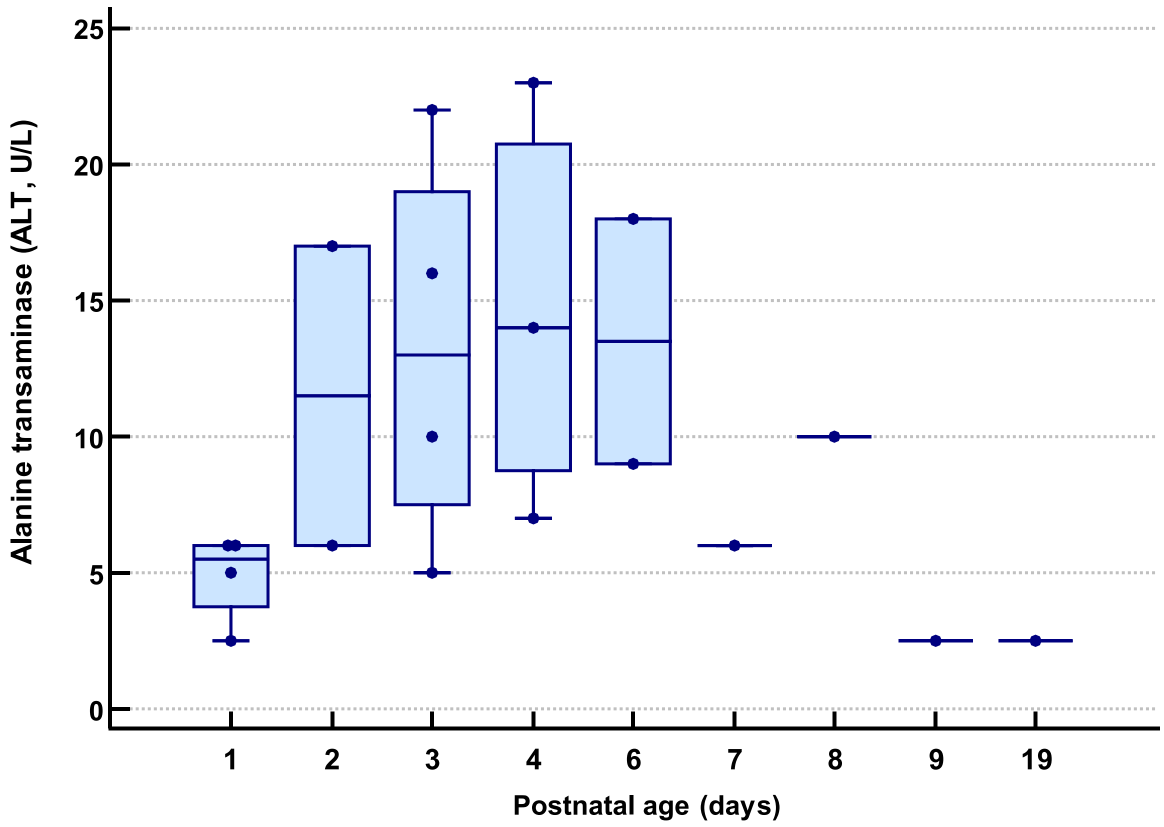

Figure S6: Available plasma alanine transaminase (ALT, U/L) data of the study population during the neonatal period (postnatal age day 1-28) are presented as boxplots. The dots represent all individual measurements. The x-axis indicates each postnatal day on which ALT observations are available. From day 7, all data originate from 1 patient. 3 ALT values were reported as below limit of quantification (LOQ) of 5, and were replaced by LOQ / 2 = 2.5

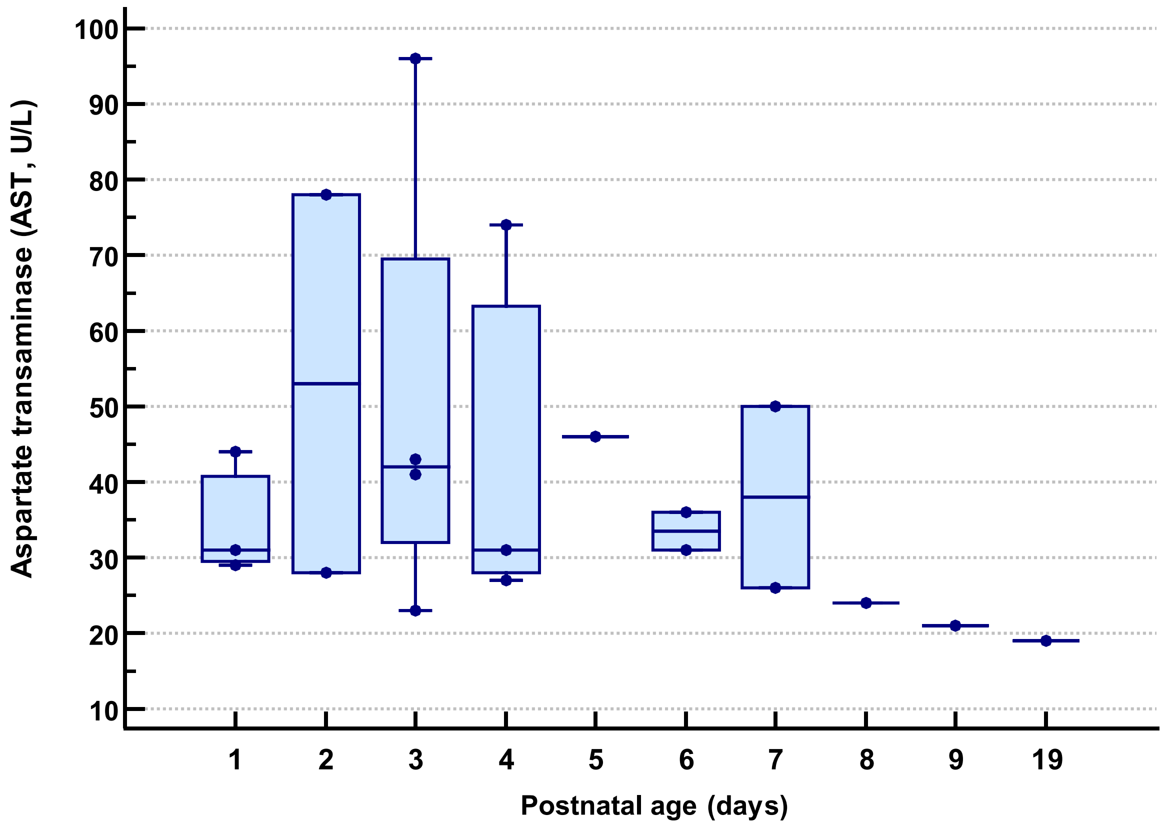

Figure S7: Available plasma aspartate transaminase (AST, U/L) data of the study population during the neonatal period (postnatal age day 1-28) are presented as boxplots. The dots represent all individual measurements. The x-axis indicates each postnatal day on which AST observations are available. From day 8, all data originate from 1 patient.

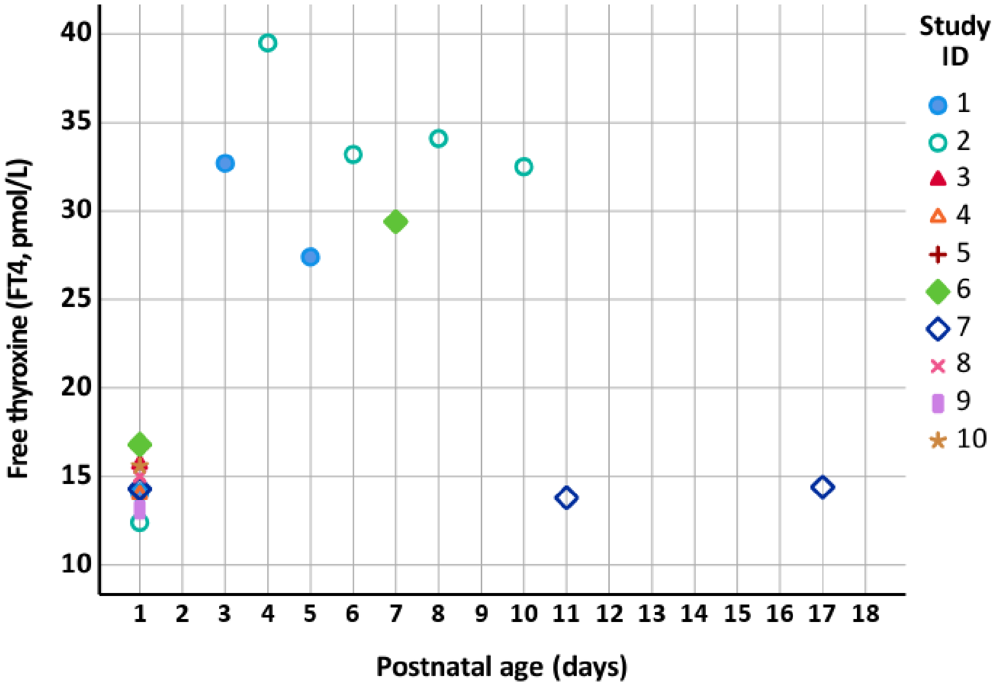

Figure S8: Available plasma free thyroxine (FT4, pmol/L) data of the study population during the neonatal period (postnatal age day 1-28) are presented. Due to the limited number of observations for each neonate, and the variable sampling dates, data are presented by a unique symbol for each individual. FT4 observations are available until postnatal age day 17. For one neonate FT4 was reported in ng/dL (2011). Conversion to pmol/L was performed as ng/dL x 12.86.

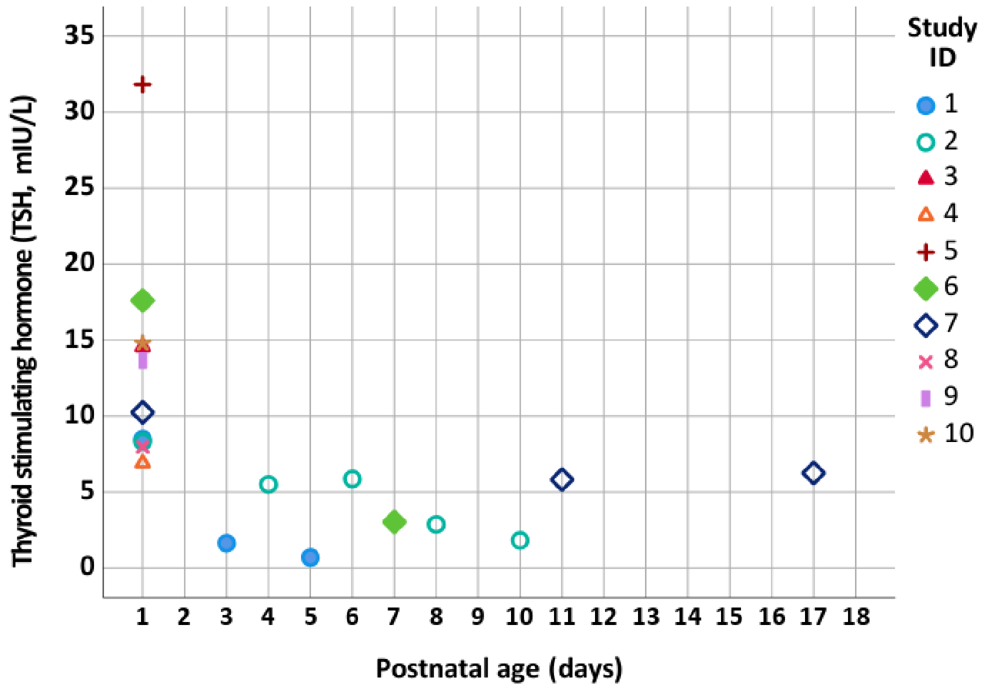

Figure S9: Available plasma thyroid stimulating hormone (TSH, mIU/L) data of the study population during the neonatal period (postnatal age day 1-28) are presented. Due to the limited number of observations for each neonate, and the variable sampling dates, data are presented by a unique symbol for each individual. TSH observations are available until postnatal age day 17.

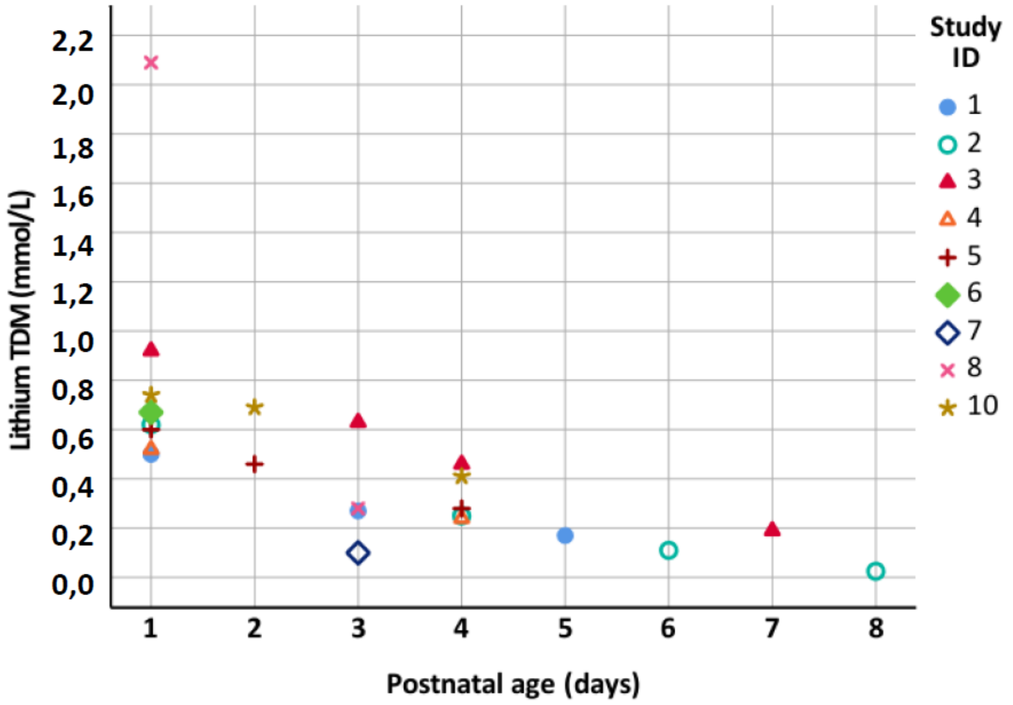

Figure S10: Available neonatal lithium serum concentrations (lithium TDM, mmol/L) are presented. Due to the limited number of observations for each neonate, and the variable sampling dates, data are presented by a unique symbol for each individual. TDM observations are available until postnatal age day 8.
